# Management of large renal stones: laparoscopic pyelolithotomy versus percutaneous nephrolithotomy

**DOI:** 10.1186/s12894-017-0266-7

**Published:** 2017-08-31

**Authors:** Yunjin Bai, Yin Tang, Lan Deng, Xiaoming Wang, Yubo Yang, Jia Wang, Ping Han

**Affiliations:** Department of Urology, Institute of Urology, West China Hospital, Sichuan University, Guoxue Xiang#37, Chengdu, Sichuan 610041 China

**Keywords:** Laparoscopic pyelolithotomy, Percutaneous nephrolithotomy, Renal stone, Meta-analysis

## Abstract

**Background:**

Percutaneous nephrolithotomy (PCNL) remains the standard procedure for large (≥2 cm) renal calculi; however, laparoscopic pyelolithotomy (LPL) can be used as an alternative management procedure. The aim of present study was to compare LPL and PCNL in terms of efficacy and safety for the management of large renal pelvic stones.

**Methods:**

A literature search was performed in Jan 2016 using electronic databases (Cochrane Central Register of Controlled Trials, Medline, and EMBASE) to identify relevant studies for the meta-analysis. Only comparative studies investigating LPL versus PCNL were included. Effect sizes were estimated by pooled odds ratio (ORs) and mean differences (MDs) with 95% confidence intervals (CIs).

**Results:**

Five randomized and nine non-randomized studies were identified for analysis, involving a total of 901 patients. Compared with PCNL, LPL provided a significantly higher stone-free rate (OR 3.94, 95% CI 2.06–7.55, *P* < 0.001), lower blood transfusion rate (OR 0.28, 95% CI 0.13–0.61, *P* = 0.001), lower bleeding rate (OR 0.20, 95% CI 0.06–0.61, *P* = 0.005), fewer hemoglobin decrease(MD -0.80, 95% CI -0.97 to −0.63, *P* < 0.001), less postoperative fever (OR 0.38, 95% CI 0.21–0.68; *P* = 0.001), and lower auxiliary procedure rate (OR 0.24, 95% CI 0.12–0.46, *P* < 0.001) and re-treatment rate (OR 0.20, 95% CI 0.07–0.55, *P* = 0.002). However, LPL had a longer operative time and hospital stay. There were no significant differences in conversion to open surgery and prolonged urine leakage rates between LPL and PCNL.

**Conclusions:**

Our present findings suggest that LPL is a safe and effective approach for management of patients with large renal stones. However, PCNL still suitable for most cases and LPL can be used as an alternative management procedure with good selection of cases.

**Electronic supplementary material:**

The online version of this article (10.1186/s12894-017-0266-7) contains supplementary material, which is available to authorized users.

## Background

Percutaneous nephrolithotomy (PCNL) currently remains the first-line treatment for large or complex renal stones. Although it is a minimally invasive procedure with higher stone-free rate (SFR), there are still serious complications [1], such as bleeding and postoperative sepsis. Size of the stone was directly correlating with the overall incidence of complications after PCNL [2]. Therefore, treatment of large renal stones is still a challenging problem in urology.

The ideal procedure for large or complex renal stones would be the one that achieve complete stone free status with minimal morbidity and with the least number of procedures. The traditional standard procedure was open nephrolithotomy, which evolved into PCNL or retrograde intrarenal surgery [3]. With the recent development of technique in laparoscopic surgery, laparoscopic pyelolithotomy (LPL) has been frequently considered as an alternative procedure in the management of large or complex renal stones to PCNL or open surgery [4]. There are some advantages to LPL, the first and most obvious advantage is that most of the stones can be removed integrally, in the next place, including the ability to minimize bleeding, lessen pain, and lower morbidity. Despite the potential advantages, its rare usage.

One prior meta-analysis [4] evaluated the efficacy and safety of LPL and PCNL in treating large renal stones and found that PCNL and LPL were effective and safe for managing this condition, but also found that LPL seems to be more advantageous. Recently, several additional clinical trials have been reported that compared PCNL and LPL for removal of large renal stones. Therefore, we perform an update meta-analysis to compare LPL and PCNL in terms of efficacy and safety for the management of large renal pelvic stones.

## Methods

### Literature search and article selection

An electronic search was performed in Jan 2016 using Medline, EMBASE, and the Cochrane Collaboration Central Register of Controlled Clinical Trials databases to identify relevant studies, using words related to percutaneous nephrolithotomy, laparoscopic pyelolithotomy, and renal calculi in all fields. Searches were restricted by English and in adult population. We also reviewed all the references of relevant articles, and recent reviews.

For studies to be included, they had to meet the following criteria: (1) patients with a large renal calculi (≥2 cm); (2) the comparison of LPL with PCNL;(3) report on at least one outcome or the data would allow the calculation; and (4) randomized controlled trial (RCT), quasi randomized controlled study, or case-control study(CCS). The most recent or complete report was used for multiple reports describing the same population. For example, when full article and conference abstract describing the same population, the former would be included. The final selection of the included studies was achieved through a consensus meeting of the reviewers.

### Data extraction and quality assessment

Two authors independently confirmed study eligibility and extracted data. Any discrepancies were resolved by discussion. The following variables were extracted from each eligible study: characteristics, interventions, and outcome measures. Our outcomes were the SFR at 12 weeks after the procedure, auxiliary procedures rate, operative time, drop in hemoglobin level, length of stay, complication rate, blood transfusion rate, and postoperative fever. The methodological quality of the studies was assessed using the Newcastle-Ottawa Scale (NOS) for non-RCTs [5] and the Jadad scale for RCTs [6].

### Data synthesis and analysis

Data analysis was performed with Review Manager version 5.1(Cochrane Collaboration, Oxford, UK). Odds ratio (OR) was applied in dichotomous outcomes, and mean difference (MD) was used for the continuous variables. Respective 95% confidence intervals (CI) were calculated for each estimate. For studies presenting continuous data as means and range, standard deviations were calculated using the methodology described by Hozo and colleagues [7]. Pooled estimates were calculated with the fixed-effect model if no significant heterogeneity was detected; otherwise, the random-effect model was used. We assessed statistical heterogeneity among studies using the chi-square test and the degree of inconsistency (I^2^). The pooled effects of OR/MD were determined by the z test, and *P* < 0.05 was considered to be statistically significant. Publication bias was evaluated by using a funnel plot.

## Results

### Study characteristics

**Fig. 1:**
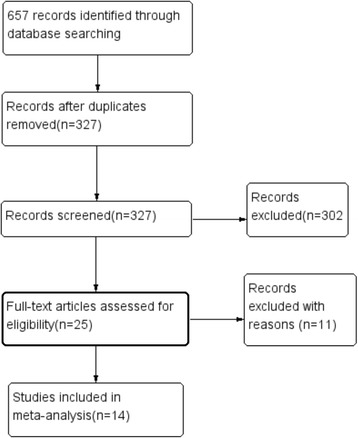
Flowchart of the studies selection process

The present study met the PRISMA statement (Additional file 1). The search identified 657 records, which were doubly screened. After study assessment, we identified 14 studies [8–21] fulfilled inclusion criteria (Fig. 1), 11 publications were full articles and three were conference abstracts [12, 13, 16]. Baseline characteristics and intervention protocols are summarized in Table 1. There were 901 patients involved in the 14 studies: 432 underwent LPL and 469 PCNL. Baseline information of study populations was comparable between LPL and PCNL groups. The types of imaging used in the studies included kidney-ureter-bladder X-ray, ultrasonography, computer tomography, fluoroscopy, and nephrostogram. There are five RCTs [8, 13–15, 18], which information on method of randomization and allocation concealment was absent or unclear. The methodological quality of included studies was relatively high for four of the CCSs and medium for four RCTs and six CCSs, whereas relatively low were found to be of two studies (Table 1).

**Table 1:** The basic characteristic of included studies

Study/year	Study design	Study period	Surgical approach	Sample size	Age (year) (mean±SD)	Staghorn(%)	Stone feature	Stone burden (mean ±SD)	Study quality
Goel 2003 [11]	CCS	1995–2003	RLP	16	38.9(21–60)	NA	Solitary, Pelvis	3.6 cm(3.2–4.5)	5^a^
			PCNL	12	41.4(20–62)	NA		4.1 cm(3.5–5.2)	
Al-Hunayan 2011 [8]	RCT	2002–2010	RLP	55	41.2 ± 11.7	NA	Solitary, Pelvis	2.4 ± 0.4 cm	3^b^
			PCNL	50	38.9 ± 11.9	NA		2.5 ± 0.4 cm	
Perlin 2011 [12]	CCS	2009–2011	RLP	5	-	NA	Solitary, Pelvis	>2.3 cm	5^a^
			PCNL	20	-	NA		>2.3 cm	
Tefekli 2012 [9]	CCS	2006–2009	RLP	26	36.5 ± 11.1	NA	Solitary, Pelvis	>4 cm^2^	6^a^
			PCNL	26	37.1 ± 10.0	NA		>4 cm^2^	
Aminsharifi 2013 [10]	CCS	2009–2012	RLP	30	43.8 ± 15.0	NA	Solitary, Pelvis	3.53 ± 0.73 cm	5^a^
			PCNL	30	45.3 ± 14.8	NA		3.66 ± 0.7 cm	
Singh 2014 [14]	RCT	2010–2012	RLP	22	45.55 ± 14.22	NA	Solitary, Pelvis	>3 cm	3^b^
			PCNL	22	44.95 ± 13.81	NA		>3 cm	
Fawzi 2015 [13]	RCT	2012–2014	RLP	30	42.4 ± 12.1	NA	Solitary, Pelvis	3.2 ± 0.6 cm	2^b^
			PCNL	30	44.6 ± 11.4	NA		3.4 ± 0.5 cm	
Basiri 2014 [15]	RCT	2009–2012	TLP	30	38.5 ± 15.9	40	Pelvis	3.6 (2.8–4.4)cm	3^b^
			PCNL	30	42.1 ± 14.3	30		3.3 (2.7–4.2)cm	
Gaur 2001 [21]	CCS	--	RLP	42	39.12(8–65)	0	Multiple, Pelvis and calyx	2.0(1.0–4.8)cm	4^a^
			PCNL	47	34.4	0		2.9(2.0–3.8)cm	
Lee 2014 [19]	CCS	2004–2011	TLP	45	56.0 ± 13.7	4.4	Multiple, Pelvis and calyx	4.93 ± 3.03 cm	6^a^
			PCNL	39	54.3 ± 13.0	28		4.63 ± 1.65 cm	
Meria 2005 [17]	CCS	1999–2004	TLP	16	42 (21–63)	NA	Solitary, Pelvis	2.5 (2.0–3.3)cm	5^a^
			PCNL	16	45 (24–69)	NA		2.6 (2.0–4.0)cm	
Li 2014 [18]	RCT	2009–2013	RLP	89	55.63 ± 10.98	17	Pelvis	2.93 ± 1.02 cm	3^b^
			PCNL	89	53.15 ± 11.54	19		3.0 ± 0.96 cm	
Tepeler 2009 [16]	CCS	2006–2008	RLP	16	41.2 ± 16.8	NA	Pelvis	8.82 ± 3.2 cm2	3^a^
			PCNL	16	43.86 ± 14.11	NA		8.49 ± 2.6 cm2	
Haggag 2013 [20]	CCS	2009–2012	RLP	10	38.8 ± 12.17	NA	Pelvis	6.5 ± 1.20 cm2	4^a^
			PCNL	42	42.03 ± 13.17	NA		4.19 ± 2.03 cm2	

*RCT* randomized controlled trial, *CCS* case controlled study, *PCNL* percutaneous nephrolithotomy, *NA* not available, *RLP* retroperitoneal laparoscopic pyelolithotomy, *TLP* transperitoneal laparoscopic pyelolithotomy

^a^ Using Newcastle-Ottawa Scale (score from 0 to 9)

^b^ Using Jadad scale (score from 0 to 5)

### Meta-analysis outcomes

#### SFR

**Fig. 2:**
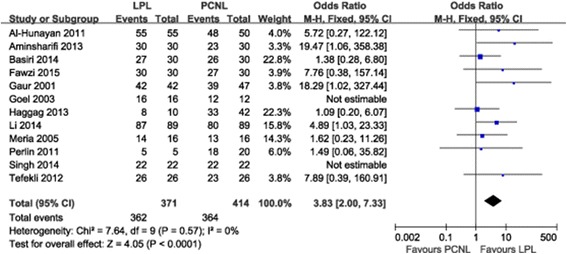
Forest plot comparing stone-free rates between two groups at 3 months after treatment. LPL, laparoscopic pyelolithotomy; PCNL, percutaneous nephrolithotomy

Two studies [16, 19] were not included in this meta-analysis as they did not assess patients’ SFR at 3 months after treatment. The pooled analysis of 12 studies showed that the SFR in the LPL and PCNL group was 97.57% (362/371) and 87.92% (364/414), respectively, and this difference was obvious (OR 3.94, 95% CI 2.06–7.55, *P* < 0.001) (Fig. 2). There was no evidence of statistical heterogeneity between studies as assessed by the Q statistic (Chi^2^ = 6.95; I^2^ = 0%).

#### Auxiliary procedures and retreatment rate

**Fig. 3:**
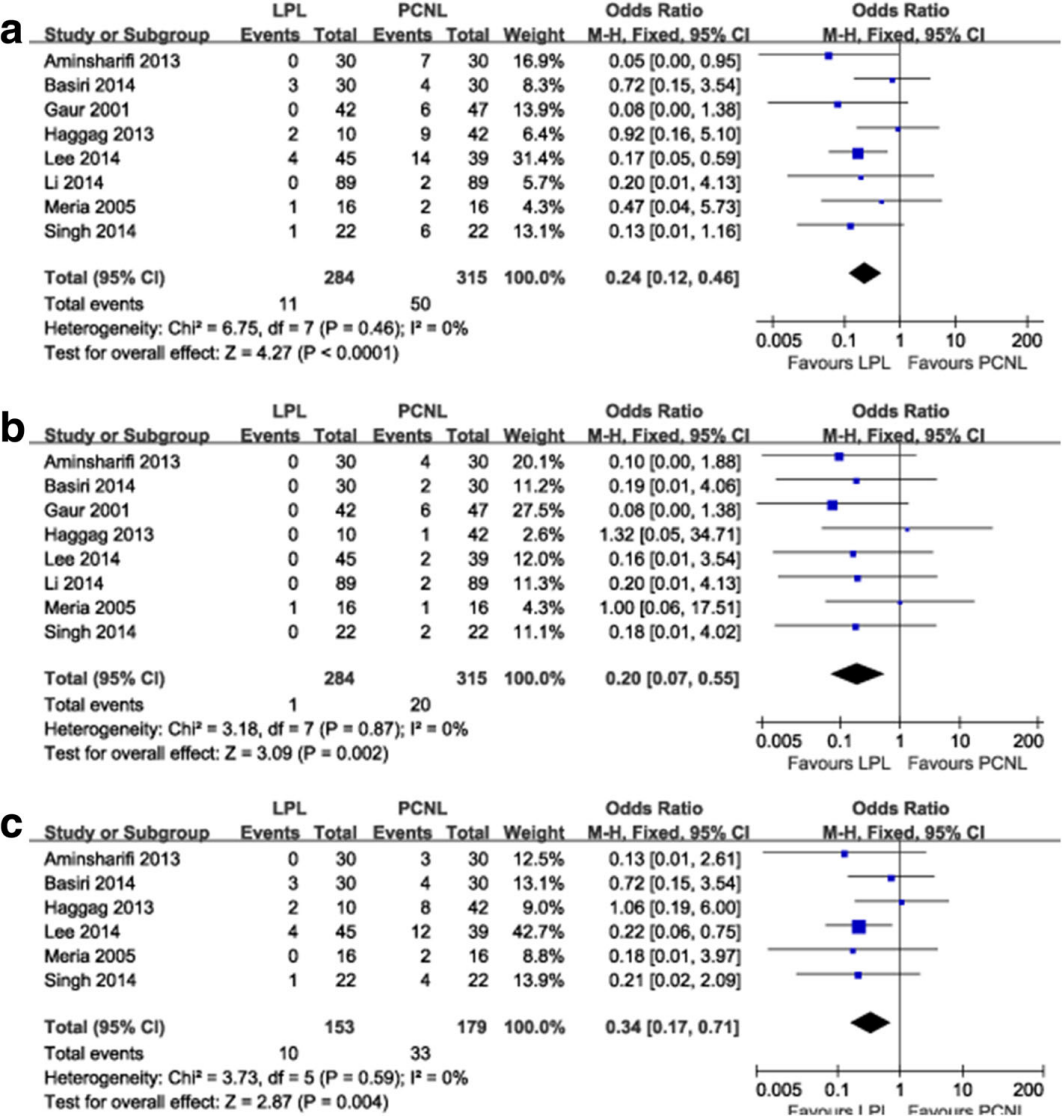
Forest plot of LPL versus PCNL: **a** auxiliary procedures rate, **b** re-treatment rate, **c** extracorporeal shockwave lithotripsy rate. LPL, laparoscopic pyelolithotomy; PCNL, percutaneous nephrolithotomy

LPL provided a significantly lower auxiliary procedures rate (OR 0.24, 95% CI 0.12–0.46, *P* < 0.001) (Fig. 3a) and lower re-treatment rate compared with PCNL (OR 0.20, 95% CI 0.07–0.55, *P* = 0.002) (Fig. 3b). The pooled analysis showed that the extracorporeal shockwave lithotripsy rate in the LPL and PCNL group was 6.54% and 18.44%, respectively, and this difference was obvious (OR 0.34, 95% CI 0.17–0.71, *P* = 0.004) (Fig. 3c).

#### Hemorrhagic complications

**Fig. 4:**
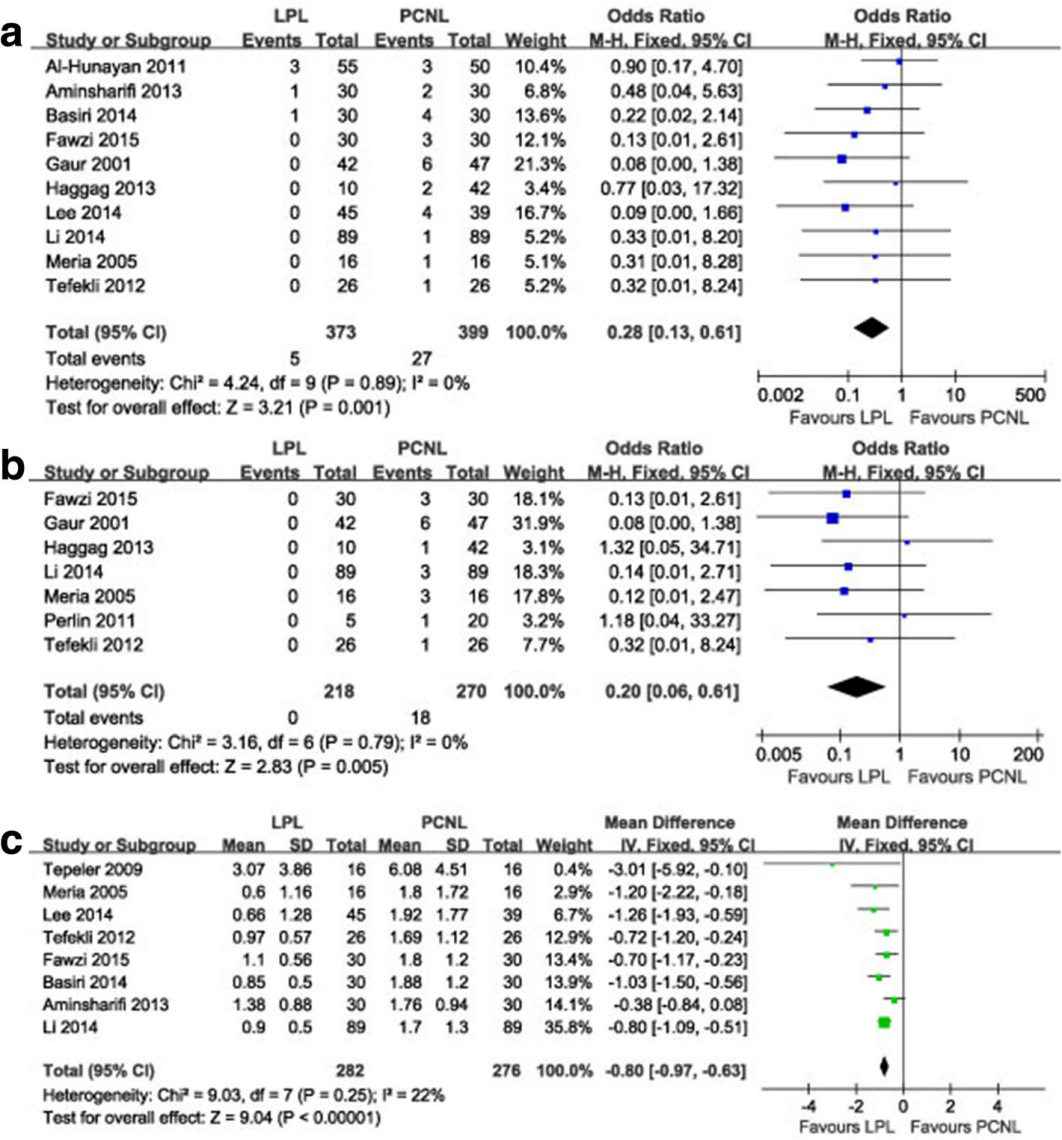
Forest plot of LPL versus PCNL: **a** blood transfusion rate, **b** bleeding rate, **c** hemoglobin decrease. LPL, laparoscopic pyelolithotomy; PCNL, percutaneous nephrolithotomy

The parameters assessed were postoperative hemoglobin drop, bleeding, and blood transfusion. LPL provided a significantly lower blood transfusion rate (OR 0.28, 95% CI 0.13–0.61, *P* = 0.001) (Fig. 4a), lower bleeding rate (OR 0.20, 95% CI 0.06–0.61, *P* = 0.005) (Fig. 4b), and fewer hemoglobin decrease (MD -0.80, 95% CI -0.97 to −0.63, *P* < 0.001) (Fig. 4c).

#### Postoperative fever and sepsis

**Fig. 5:**
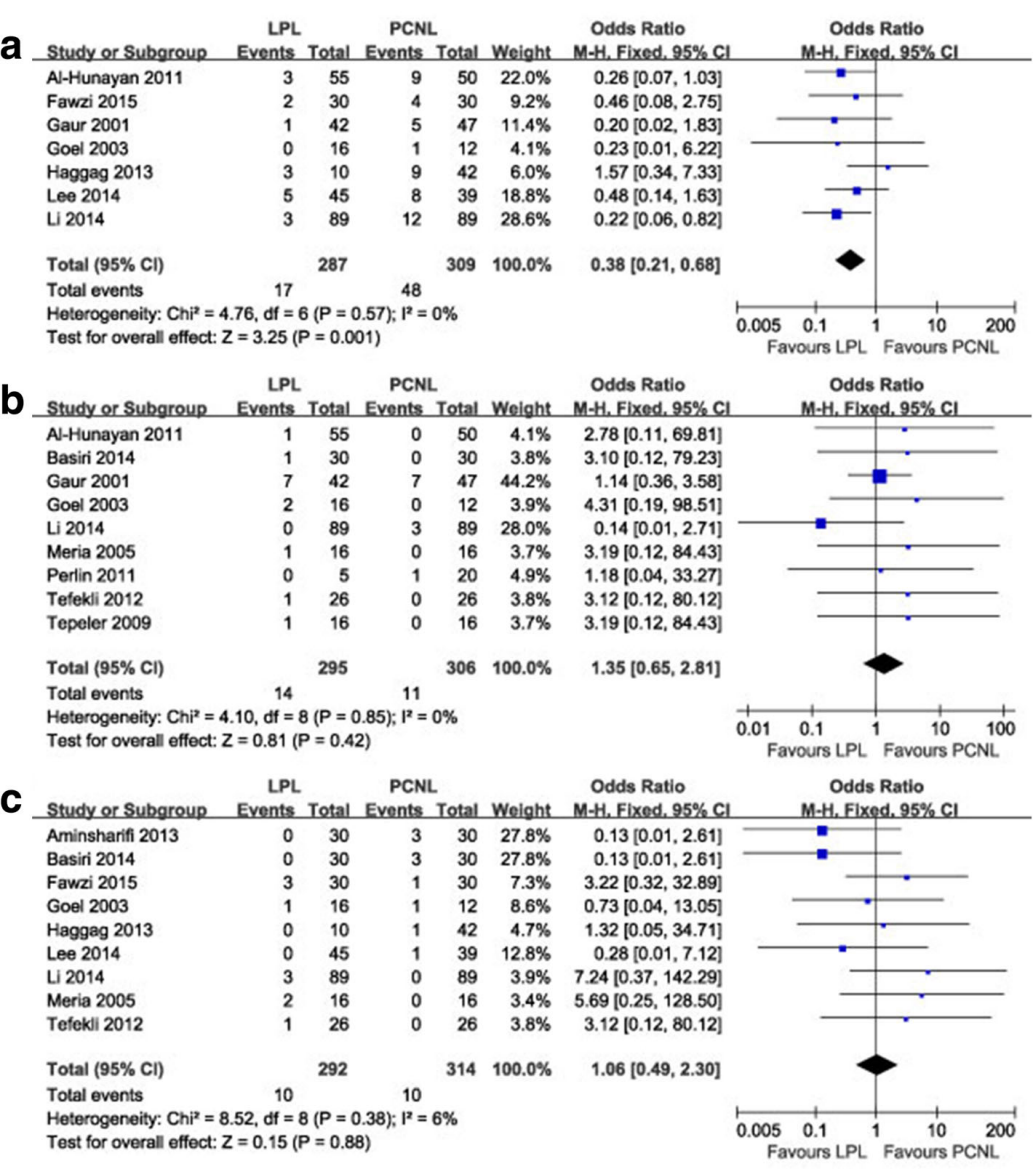
Forest plot of LPL versus PCNL: **a** postoperative fever, **b** conversion rate, **c** prolonged urine leakage. LPL, laparoscopic pyelolithotomy; PCNL, percutaneous nephrolithotomy

There were seven studies comprising 596 patients included in the meta-analysis for postoperative fever. We found that the incidence of postoperative fever was lower in the LPL group than in the PCNL group (OR 0.38, 95% CI 0.21–0.68, *P* = 0.001) (Fig. 5a) with no heterogeneity between studies (I^2^ = 0%). None of patients from the included studies encountered sepsis or septic shock after the both procedures.

#### Conversion rate and prolonged urine leakage

There was no significant difference between the LPL and PCNL groups in terms of conversion rate (OR 1.35, 95% CI 0.65–2.81, *P* = 0.42) (Fig. 5b) and the incidence of prolonged urine leakage (OR 1.06, 95% CI 0.49–2.30, *P* = 0.88) (Fig. 5c). The reasons of conversion to open surgery included injury of renal vein [15] and peritoneum [21], uncontrolled bleeding [8, 18], stone migration into the calyx [11], and perirenal adhesions [11, 16, 17].

#### Operative time and hospital stay

Operative time was reported in all included studies. Heterogeneity was observed in the pooled analysis (Chi^2^ = 250.73; I^2^ = 95%). Meta-analysis of data showed that PCNL had significantly shorter operative time than LPL (random-effect model; MD 32.86, 95% CI 12.85–52.86, *P* = 0.001). Sensitivity analysis was performed by sequentially removing each study, whereas this substantively did not affect the result. This result indicated that the meta-analysis was not influenced by any one study.

Thirteen studies reported the length of hospital. The results of meta-analysis of these studies indicated a benefit of shorter length of hospital stay in the PCNL group (random-effect model; MD 0.33, CI 95% -0.24 to 0.89, *P* = 0.002) with significant heterogeneity between studies (Chi^2^ = 87.37; I^2^ = 86%). We could not identify any plausible cause by sensitivity analysis.

#### Subgroup analyses

The subgroup analyses suggested that the results of this meta-analysis were relatively stable. When the studies included patients with intra-renal pelvis and multiple stones (≥2) were removed [15, 16, 18–21], most of the outcomes including SFR (OR 5.10, 95% CI 1.79–14.51, *P* = 0.002, I^2^ = 0%), auxiliary procedures rate (OR 0.13, 95% CI 0.03–0.52, *P* = 0.004, I^2^ = 0%), conversion rate (OR 2.82, 95% CI 0.70–11.46, *P* = 0.15, I^2^ = 0%), the incidence of prolonged urine leakage (OR 1.27, 95% CI 0.45–3.61, *P* = 0.65, I^2^ = 4%), level of hemoglobin decrease(MD -0.71, 95% CI -0.90 to −0.51, *P* < 0.0001, I^2^ = 0%), bleeding rate (OR 0.23, 95% CI 0.05–1.05, *P* = 0.06, I^2^ = 0%), blood transfusion rate(OR 0.44, 95% CI 0.16–1.26, *P* = 0.13, I^2^ = 0%), postoperative fever rate (OR 0.31, 95% CI 0.11–0.87, *P* = 0.03, I^2^ = 0%), operative time (random-effect model; MD 39.39, 95% CI 21.33–57.45, *P* < 0.0001, I^2^ = 87%), and hospital stay (random-effect model; MD 0.74, CI 95% 0.29–1.18, *P* = 0.001, I^2^ = 44%) were not greatly affected.

#### Publication bias analyses

We analyzed possible publication bias by generating funnel plots of the studies used for all of the evaluated comparisons of outcomes. No significant publication bias was observed in the above-mentioned analyses.

## Discussion

One previous meta-analysis [4] included seven studies with 176 patients underwent LPL and 187 PCNL, showed equivalency for conversion rate, blood transfusion, prolonged urine leakage, and found higher SFR and lower incidence of bleeding and postoperative fever in the LPL group than PCNL group. In addition, the results of the previous study showed that operative time and length of hospital stay were shorter in the PCNL group, drop in hemoglobin level was fewer in the LPL group. In present study, we included 14 studies involving 432 patients underwent LPL and 469 PCNL, and found similar results from the previous meta-analysis regarding SFR, conversion rate, operative time, length of hospital stay, hemoglobin decrease, and postoperative fever. However, in term of blood transfusion rate, we found that there was a significantly lower blood transfusion rate in the LPL group than in the PCNL group, which was different to the previous meta-analysis result. We also found that LPL provided a significantly lower auxiliary procedures and re-treatment rate. The main reason for this difference might be due to the different sample sizes between previous and present studies, which also was the reason of our performed the present study.

Although the SFR was assessed in a different way in each study, the result revealed LPL provided a statistically higher SFR at 3 months after treatment than PCNL, regardless of the definition. The reason may be that most of the stones can be removed integrally in LPL. In the PCNL group, disintegration of the stone may have left some residual stones which can form nuclei for stone recurrence, and the scattering of stone fragments may reduce success rates, which associated with a significantly higher auxiliary procedures and re-treatment rate than LPL. Currently, PCNL is the recommended treatment option for patients with staghorn calculi. However, SFR after PCNL for staghorn calculi only ranges between 49 and 78% [22]. It is noteworthy that LPL can be considered an alternative and feasible technique to PCNL for patients with complex and large renal stones. Gandhi et al. [23] reported the 49 patients with staghorn stones (>3–4 cm) underwent LPL, the mean SFR in one session was 90% with lower complications, no blood transfusion and only two patients had urine leak (Clavien-Dindo grade IIIa). However, the leak stopped after 10 days in both patients.

Our results showed that operative time was significantly shorter for PCNL than LPL. As known to all, the operative time is directly related with many variables such as the types of approach, surgeon’s experience, individual differences of patients, and the different equipment used. In LPL procedure, closure of the pyelotomy incision requires advanced laparoscopic skills. Sometimes, delicate renal pelvis tissues, always caused by long-term chronic inflammation, brings many challenges for the closure of the pyelotomy incision [17] and prolongs the operative time. The longer time of LPL was usually related to the long learning curve as well as the time needed for intracorporeal suturing and delivery of the stone into the endobag [8]. However, Li et al. [18] randomized 178 patients with large renal pelvis stones into two groups found the mean operative time was significantly shorter in the LPL than PCNL, which is likely due to stones in LPL can be removed integrally. Indeed, retrieve stones is one of the major limitations of LPL. Lee et al. [19] used a flexible nephroscope to overcome this difficulty which enable easier approach. With the development of robot technology in urology, this interface maybe will improve the limits of tissue dissection, stone extraction during laparoscopy, intracorporeal reconstruction, and suturing, thereby having the potential to improve the outcomes and flattening the learning curve. However, much less is known about the relative outcomes and costs in robot-assisted pyelolithotomy, which is a major consideration in robotic surgery.

Although LPL have a longer operation time, this may be compensated by the lower complication and higher SFR. Postoperative fever secondary to an urinary tract infection (UTI) in patients with PCNL ranges between 2.8 and 32.1% [1]. Kidney stones are foreign bodies of the urinary tract and can allow bacteria to grow onto them and then become a reservoir for bacteria. They are disintegrated, bacteria are released from the stone into the collecting system, which tends to result in bacteriuria, bacteremia, and clinical UTI. Recently study demonstrated residual stone is a major contributing factor for the development of fever after tubeless PCNL [24]. This finding may translate into a clinical benefit for the patients in that stones removed integrally or the higher SFR of the LPL was associated to lower incidence of postoperative fever. Septic shock, the incidence after PCNL was 2.4% [25], is one of the most dangerous complications after lithotomy due to it can lead to significant mortality. The risk factors for septic shock includes positive urine culture, female gender, renal insufficiency, diabetes mellitus, high pressure of irrigation fluid during PCNL, staghorn calculus, infected stones, indwelling catheters, obstruction, and duration of the operation (> 90 min) [25, 26]. Positively, strict control of blood glucose and pre-operation antibiotics used could reduce the possibility of post-PCNL septic shock. Early recognition and timely comprehensive treatment of septic shock may decrease the mortality. In addition, infective or septic complications may be associated with laparoscopic approach. Transperitoneal approach could be more at risk about it due to this approach might lead to increase the interference of the abdominal organs, postoperative intra-abdominal infection, and the possibility of adhesions. Further prospective randomized controlled trials are needed to determine which approach should be favored.

Although LPL appears to be more invasive because three or four trocar punctures are needed compared with PCNL in which only a single percutaneous access was made, PCNL make renal parenchymal more susceptible to injury with it tends to result in various complications, such as nephron damage and bleeding. Bleeding after PCNL is common, which leads to a more frequent use of blood transfusion, according to previous reports, 1–12% of patients require [1]. With increasing stone burden, patients with PCNL, not only SFR decreased, but also the risk of blood transfusion increased [2]. Risk factors for severe bleeding were upper pole access, solitary kidney, staghorn stone, multiple punctures and inexperienced surgeon [27]. Therefore, PCNL should be performed by an experienced surgeon in patients at risk for severe bleeding. On the other hand, these patients might as well choose other alternative procedures. Our study found that LPL provided a significantly lower blood transfusion rate, lower bleeding rate, and fewer hemoglobin decrease. The reason was probably due to the fact that LPL harmlessness for renal parenchyma. Whatever the approaches, patients with bleeding tendencies needs careful preoperative, intra- and post-operative management because both the procedures may lead to a kidney loss.

For conversion rate and prolonged urine leakage, regardless of our or previous meta-analyses, the results were similar between the two groups. However, more incidence of prolonged urine leakage and longer hospital stay were found in the LPL group. Urine leakage can be attributed to incomplete closure of the pyelotomy incision after LPL, which can prolong hospital stay [8]. Closure of pyelotomy incision is technically difficult during laparoscopic surgery, advanced experience and high skills or robot-assisted surgery are needed. But urinary leakage has been minimized with advances in intracorporeal suturing techniques, such as barbed suture [28]. In addition, suture time was significantly decreased with barbed suture use during laparoscopic pyeloplasty [29], hence, this will shorten operative time.

However, the complications can be minimized with proper patient selection and sufficient preoperative preparation. LPL is certainly safe and feasible in experienced hands, but should not replace PCNL, which remains the gold standard for kidney stones greater than 2 cm. These procedures are technically challenging and should only be performed by experienced laparoscopic surgeons. According previous studies, LPL is more suitable for patients with urinary deformity require concomitant pyeloplasty.. Patients with previous history of open renal surgery always have significant perinephric adhesion which may affect the success or complication rate in LPL, does not in PCNL [30]. Therefore, PCNL is the first-selected treatment in such situation. LPL cannot be a feasible modality for renal stones with intrarenal pelvis, which increased the incidence of prolonged urine leakage. All in all, LPL is considered a successful alternative therapy for PCNL in selected cases with large renal stones like those in the extrarenal pelvis in patients without a history of previous surgery. In addition, LPL can be considered as a reasonable therapeutic option for large staghorn calculus which cannot be removed with a reasonable number of access and sessions of PCNL.

This study has some limitations. First, the present analysis was conducted using the currently available comparative studies. However, most of the studies were CCS, had a small sample number and quality ranged from low to moderate. Second, heterogeneity among studies was found to be high for several parameters. This heterogeneity can be explained by the difference in surgical practices, patient inclusion criteria, surgeons’ experience, outcome definitions and standards. Third, the analysis did not incorporate stone shape and composition into the assessment, and either of these could have introduced bias into the analysis. Because of the above limitations might influence the interpretation of our findings, it highlights that large scale, multicenter RCTs are needed for a further robust conclusion.

## Conclusion

Our present findings suggest that LPL is a safe and effective approach for management of patients with large renal pelvic stones with the merits of higher SFR, less blood loss, and lower auxiliary procedures rate. However, PCNL still suitable for most cases and LPL can be used as an alternative management procedure with good selection of cases.

## Additional file

Additional file 1: PRISMA 2009 checklist. (DOCX 26 kb)

## Abbreviations

CCS: Case-control study
CI: Confidence interval
LPL: Laparoscopic pyelolithotomy
MD: Mean difference
NOS: Newcastle-Ottawa Scale
OR: Odds ratio
PCNL: Percutaneous nephrolithotomy
RCT: Randomized controlled trial
SFR: Stone-free rate
UTI: Urinary tract infection
